# Supplementary material for: Performance of Imidazoquinoline Glycoconjugate BAIT628 as a TLR7 Agonist Prodrug for Prostate Cancer
Source: Pharmaceuticals (Basel). 2025 May 27;18(6):804. doi: 10.3390/ph18060804 (PMC12195845; doi:10.3390/ph18060804)
Supplement: Supplementary file 1 [file pharmaceuticals-18-00804-s001.zip › pharmaceuticals-3607623-supplementary.pdf]

Supplementary Materials

Performance of imidazoquinoline glycoconjugate BAIT628 as a TLR7 agonist prodrug for prostate cancer

Seyedeh A. Najibi [1], S M Al Muied Pranto[1], Muhammad Haroon[1], Amy E. Nielsen [2], Rock J. Mancini [1,2,*]

[1] Department of Chemistry and Biochemistry, Miami University, 651 E. High Street, Oxford, OH 45056, USA

[2] Astante Therapeutics Inc., 201 E. Fifth Street, Cincinnati, OH, 45202, USA

* Corresponding Author:

Rock J Mancini: mancinr@miamioh.edu

Biological Materials

Antibody	Source	Dilution Factor
Fc Block CD16/CD32	Biolegend	1.0 ug/million cells million in 100 uL Volume
PE anti-mouse CD4 Antibody	Biolegend	0.25 ug/million cells in 100 uL Volume
PE Rat IgG2b, κ Isotype Ctrl Antibody	Biolegend	0.25 ug/million cells in 100 uL Volume
APC-Cy™7 Hamster Anti-Mouse CD11c	BDbiosciences	0.25 ug/million cells in 100 uL Volume
APC-Cy™7 Hamster IgG1, λ1 Isotype Control	BDbiosciences	0.25 ug/million cells in 100 uL Volume
CD8a Monoclonal Antibody (53-6.7), Alexa Fluor™ 488, eBioscience™	Thermofischer	0.5 ug/million cells in 100 uL Volume
Mouse IgG2a kappa Isotype Control (eBM2a), Alexa Fluor™ 488, eBioscience™	Thermofischer	0.5 ug/million cells in 100 uL Volume

Table S1: Antibodies used in this study for intratumoral staining of CD4/CD8 and CD11c populations.

Cell line	Source	Purchase Date	Biological Sex
TRAMP-C2 (CRL-2731)	ATCC	May 14, 2023	Male
RAW-Blue	Invivogen	March 19, 2023	Male

Table S2: Detailed information of the cell lines used in the study

Synthesis of BAIT628

The synthesis of BAIT628 was accomplished in 7 steps (**Scheme S1**). Although the synthesis was already reported by our group in a previous study,[1] we have modified the final global deprotection step which dramatically increased the yield of final compound 7 (BAIT628) from 42% to 63%. Detail of improved synthesis of compound 7 is given as; In a 25 mL of dry round bottom flask containing dry magnetic stir bar, 33 mg of KCN (2 equivalent) dissolved in 5 mL of dry methanol and stir 10 minutes prior to addition of compound 6 (0.2 g, 0.26 mmol). After 5 h stirring at room temperature methanol was evaporated with rotavap and obtained solid was dissolved in acetonitrile and purified with reverse phase HPLC using a water:ACN gradient. ACN was removed from the resulting purified product in vacuo before drying via lyophilization to obtain the product as a white solid.

White fluffy solid; Yield: 50%; R_f= 0.17 (10% MeOH in DCM); ^{1}H NMR (DMSO-d6)= 0.86 (d, 6H, 2x-CH_3, *J*= 6.4 Hz), 2.13 (sept, 1H, -CH, *J*= 6.4 Hz), 3.58-3.29 (m, 5H, -CH, -CH, -CH, -CH_2), 3.76 (s, 1H, -CH), 4.43-4.39 (m, 3H, -OH, -CH_2), 4.76 (d, 1H, -OH, *J*= 5.8 Hz), 4.83 (d, 1H, -OH, *J*= 5.4 Hz), 5.07 (d, 1H, -OH, *J*= 4.48 Hz), 5.14 (s, 2H, -CH_2), 5.54 (s, 1H, -CH), 7.49 (d, 1H, Ar-H, *J*= 8.8 Hz), 7.55 (t, 1H, Ar-H, *J*= 7.5 Hz), 7.60 (t, 1H, Ar-H, *J*= 6.2 Hz), 7.66 (dd, 1H, Ar-H, *J*= 1.5, 8.7 Hz), 7.90 (d, 1H, Ar-H, *J*= 8.0 Hz), 7.96 (d, 1H, Ar-H, *J*= 1.1 Hz), 8.18 (d, 1H, Ar-H, *J*= 7.8 Hz), 8.28 (s, 1H, Ar-H), 9.99 (s, 1H, -OCONH); ^{13}C NMR (DMSO-d6)= 19.8 (2x-CH_3), 28.9 (-CH), 53.9 (-CH_2), 61.4 (-CH_2), 64.9 (-CH_2), 66.8, 70.2, 70.8, 76.2, 99.9 (mannose-C), 117.2, 118.3, 121.2, 124.5, 125.9, 127.8, 129.3, 131.2, 131.6, 133.8, 134.0, 140.5, 143.4, 144.8, 145.2, 148.7 (Ar-C), 153.2 (-C=O).

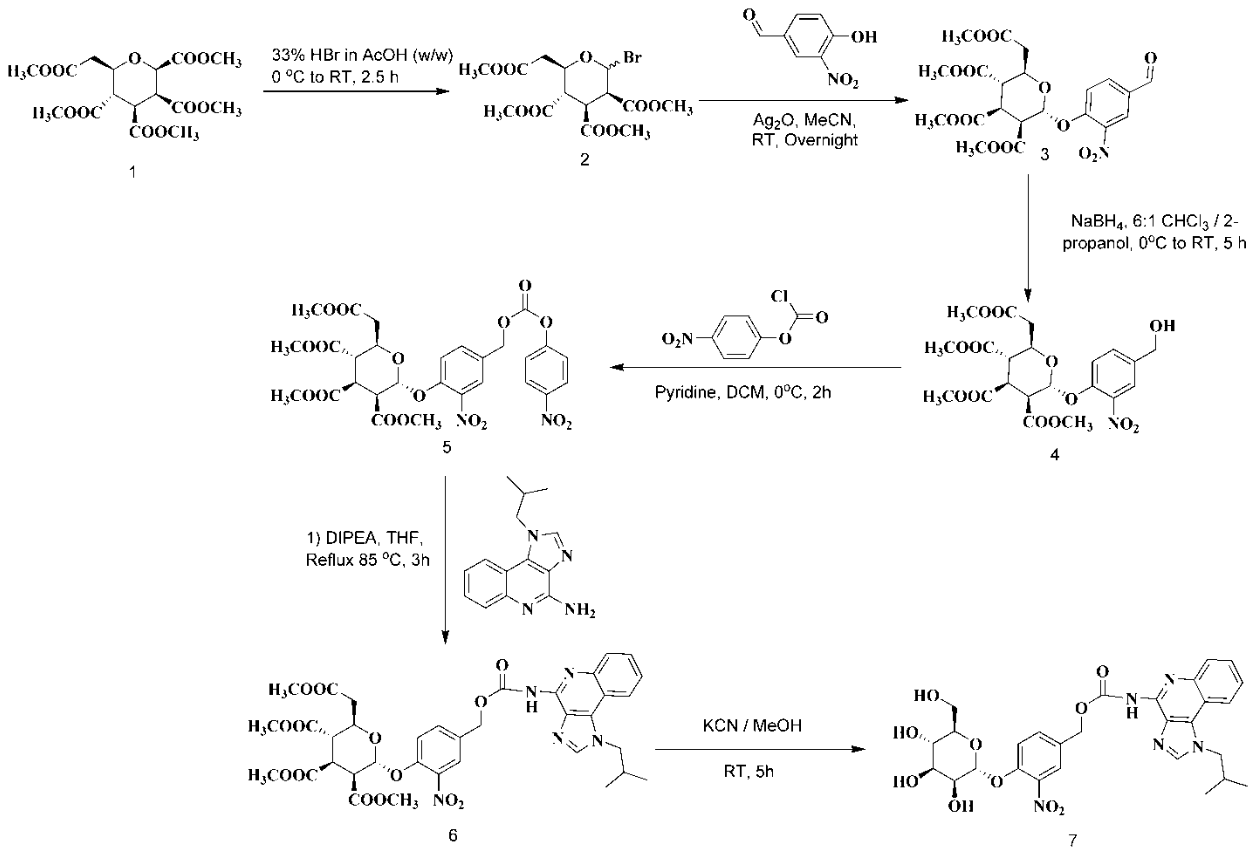

Scheme S1. Synthesis of BAIT628 previously accomplished, using modified Zemplen deacetylation conditions (MeONa / MeOH) to produce the final product (7) from (6) in modest yields typically near 40%. Using the revised conditions, yields of over 60% are routinely obtained.

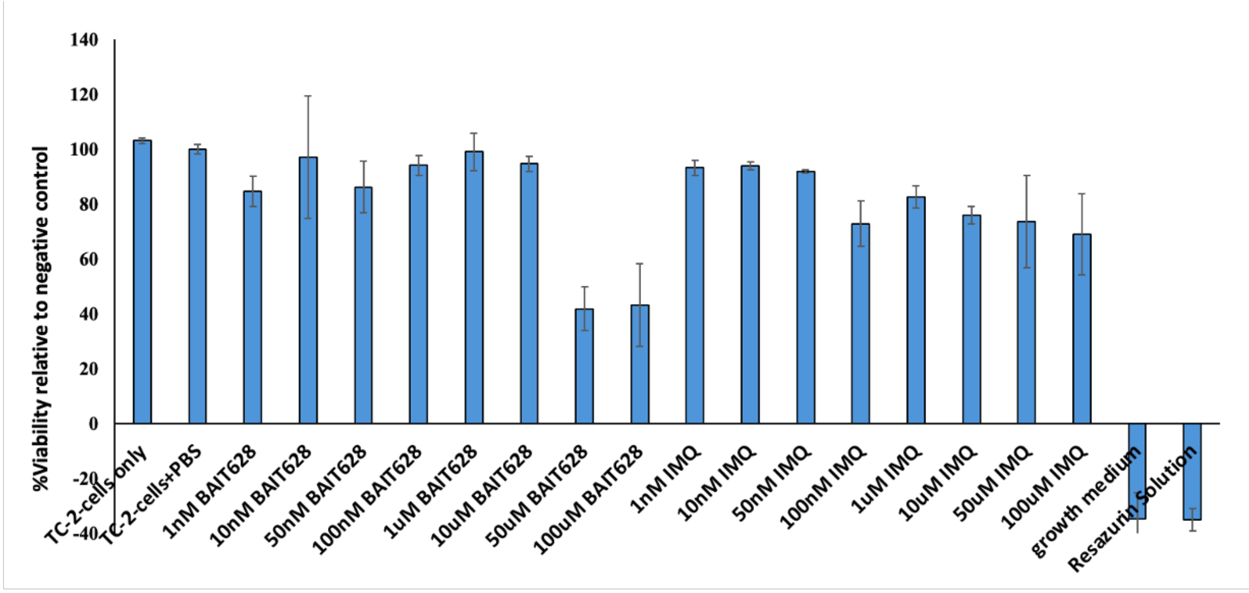

Figure S1. BAIT628 cytotoxicity on TRAMP-C2 cells. Cells were seeded at a density of a 20,000 cells/well in a 96-well plate using complete media and incubated overnight before treatment. Cells were treated with synthesized compounds for 24 h to assess cell viability. The growth medium changed with culture medium without phenolphthalein indicator and Resazurin solution (10% of the culture volume) was added. The relative absorbance was measured to assess cell viability. PBS was used as a negative control. Data are presented as the mean ±SD from three independent experiments.

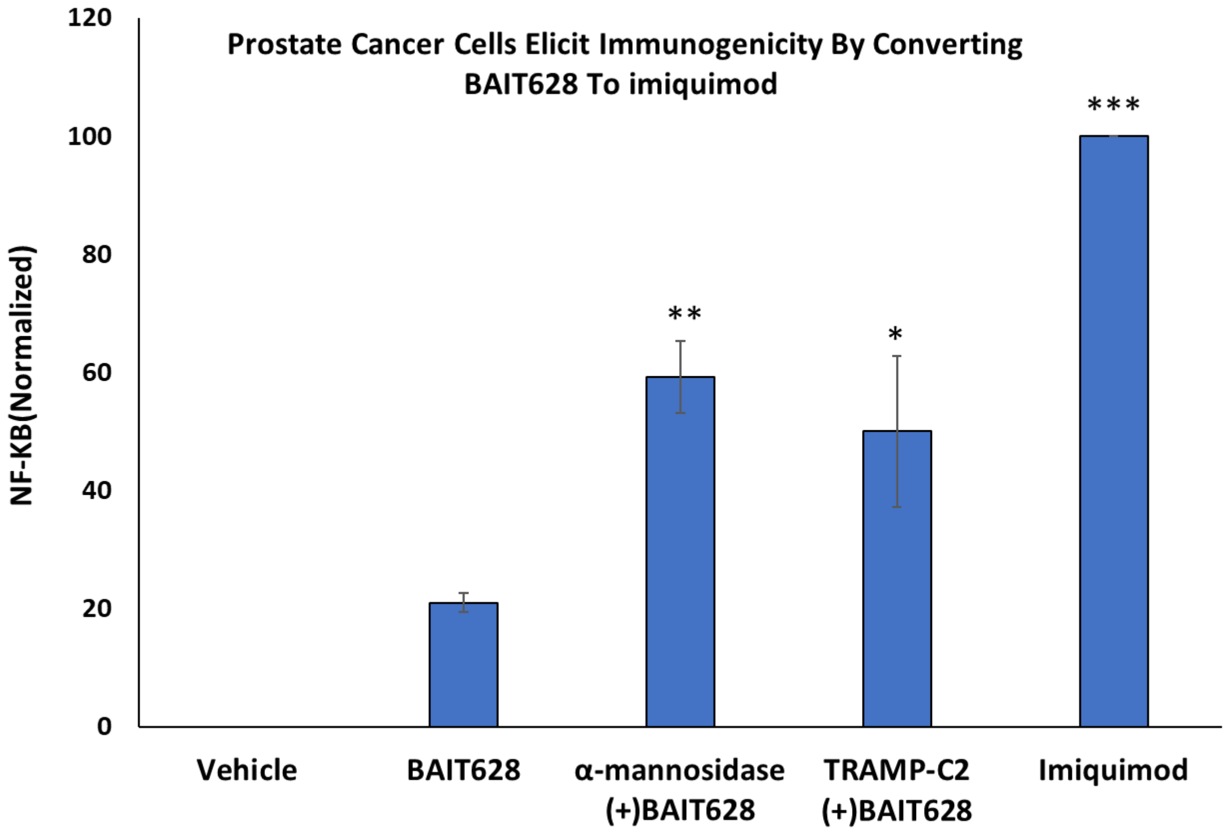

Figure S2. Activation of RAW-Blue cells following incubation of BAIT628 with TRAMP-C2 cells, RAW-Blue cells, and 0.1 U mL−1 exogenous α-mannosidase. Data is normalized relative to RAW-Blue cells treated with 1 μM Imiquimod as positive control. Error bars show standard deviation from the mean of experiments repeated in triplicate. $^*p < 0.05$, $^{**}p < 0.01$, $^{***}p < 0.001$ for experimental condition relative to BAIT628 alone.

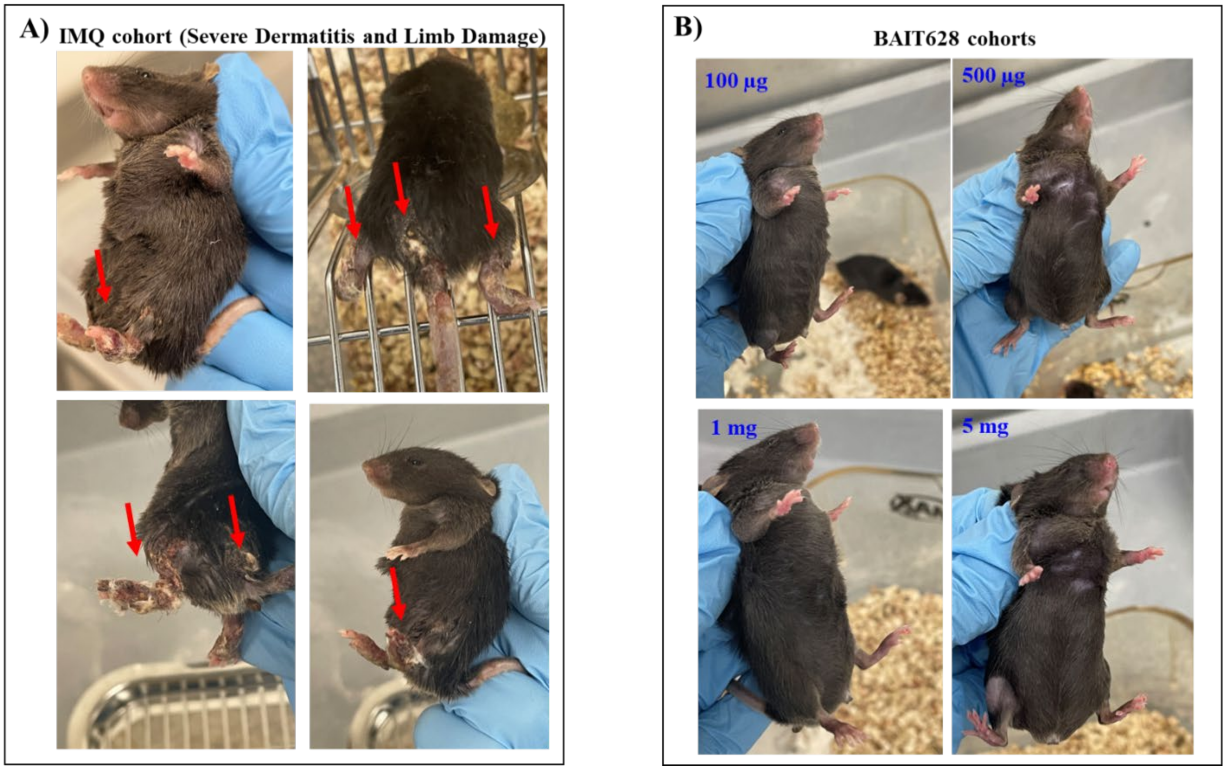

Figure S3. Body condition of mice. **A**) Mice receiving IP Imiquimod developed severe dermatitis (red arrows) and in several cases lost limbs or otherwise developed conditions which met criteria for a humane study end-point. **B**) In contrast, mice receiving BAIT628 maintained a normal body condition.

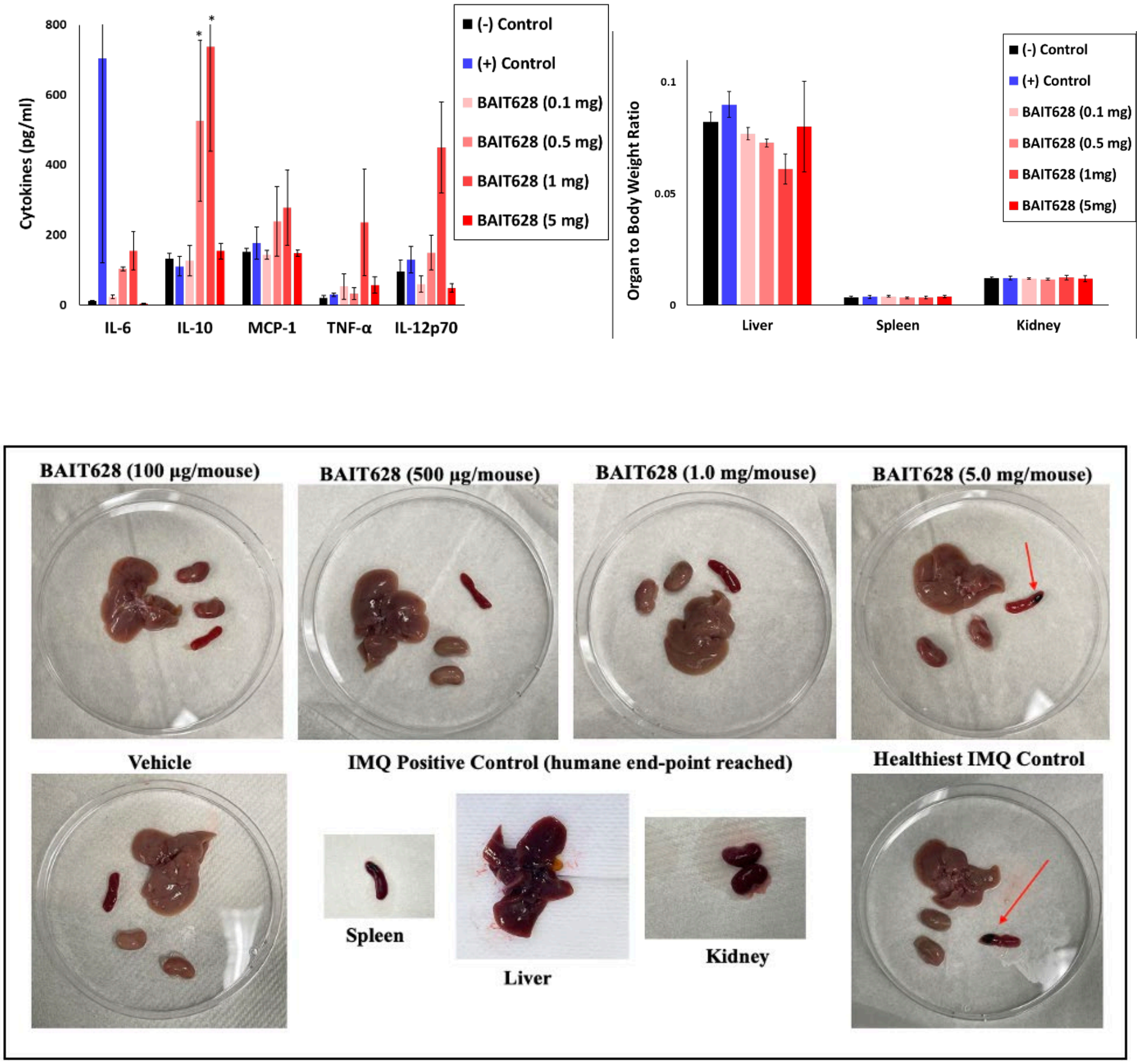

Figure S4. Organs including liver, spleen, and kidney were examined in all mice following the MTD study. Some damage was found on the spleen of mice in the highest dosed BAIT628 cohort (blue arrow), but otherwise organs from all other BAIT628 cohorts appeared healthy. Some IMQ (Positive control) mice required euthanasia prior to the study end-point and even those who survived to the end of the study also exhibited significant signs of organ damage (red arrows). Images are representative of each cohort (n = 6). Images were obtained using a digital camera. No post-acquisition manipulation was performed apart from cropping / scaling the images and adding the indicator arrows. Overall, production of IL-6 (IMQ) or lack of IL-10 production (BAIT628 5mg / mL) appeared to correlate with organ pathology.

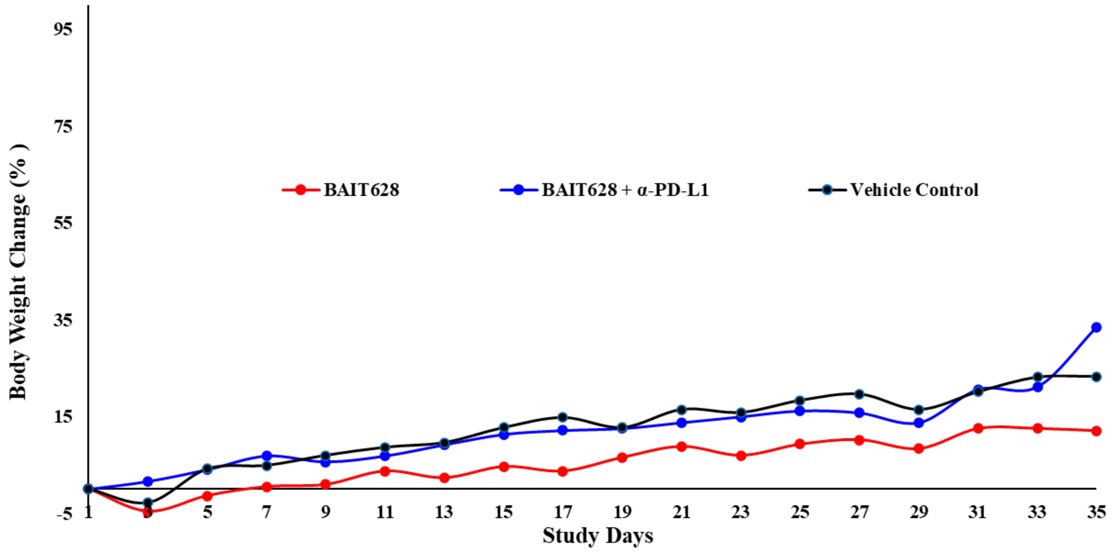

Figure S5. Mean body weight change of mice for the duration of the study. There was not a significant difference in body weight between the study groups.

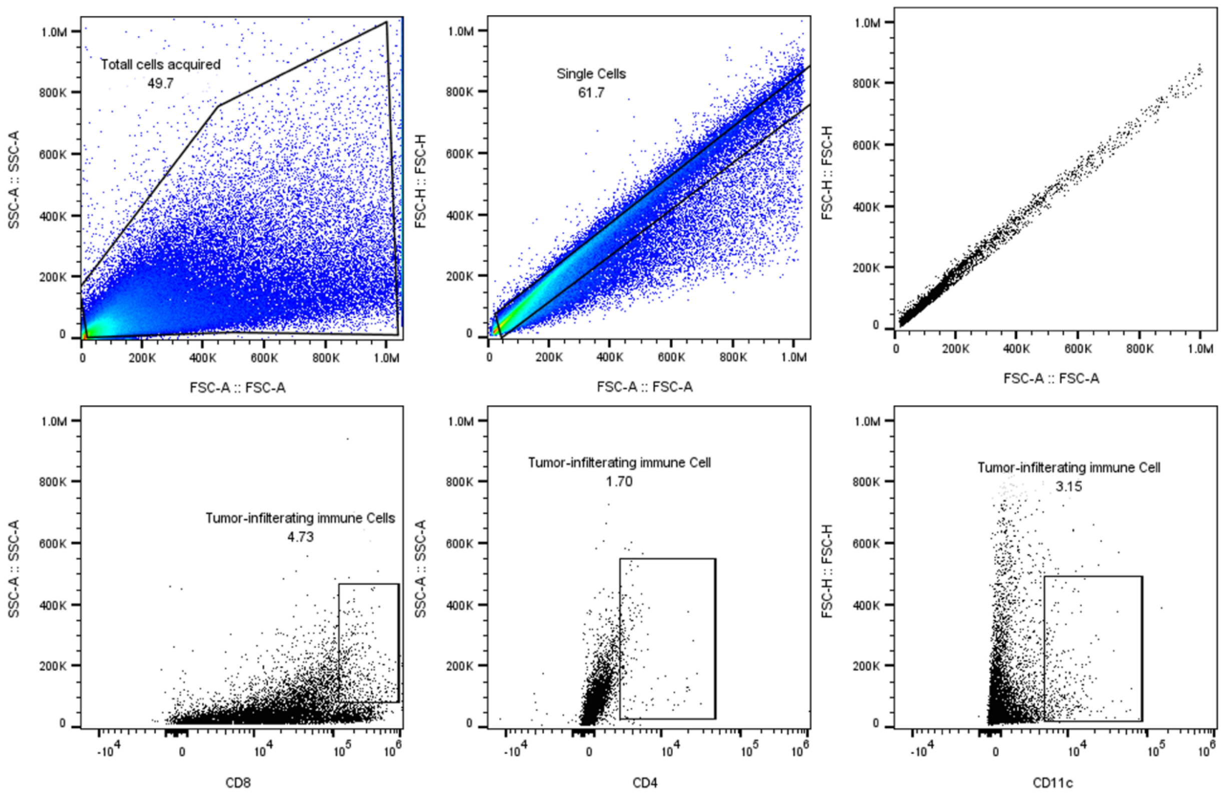

Figure S6. A representative Schema of gating strategy for flow cytometry data acquisition. Gating Strategy illustrating lymphocyte population being subgated to the level of CD11c+ dendritic cells, CD4+ and CD8+ T Cells.

SSC-A: side scatter-area

FSC-H: forward scatter – height

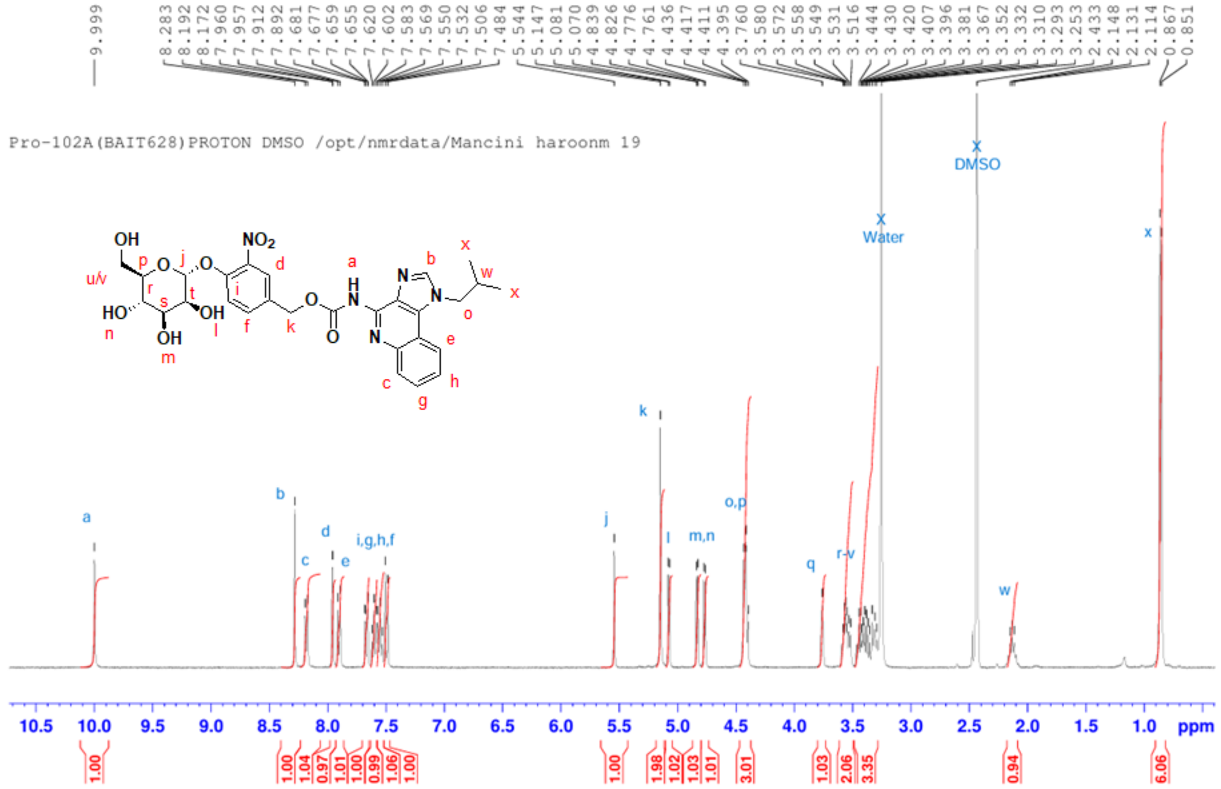

Figure S7: ^{1}H-NMR of BAIT 628

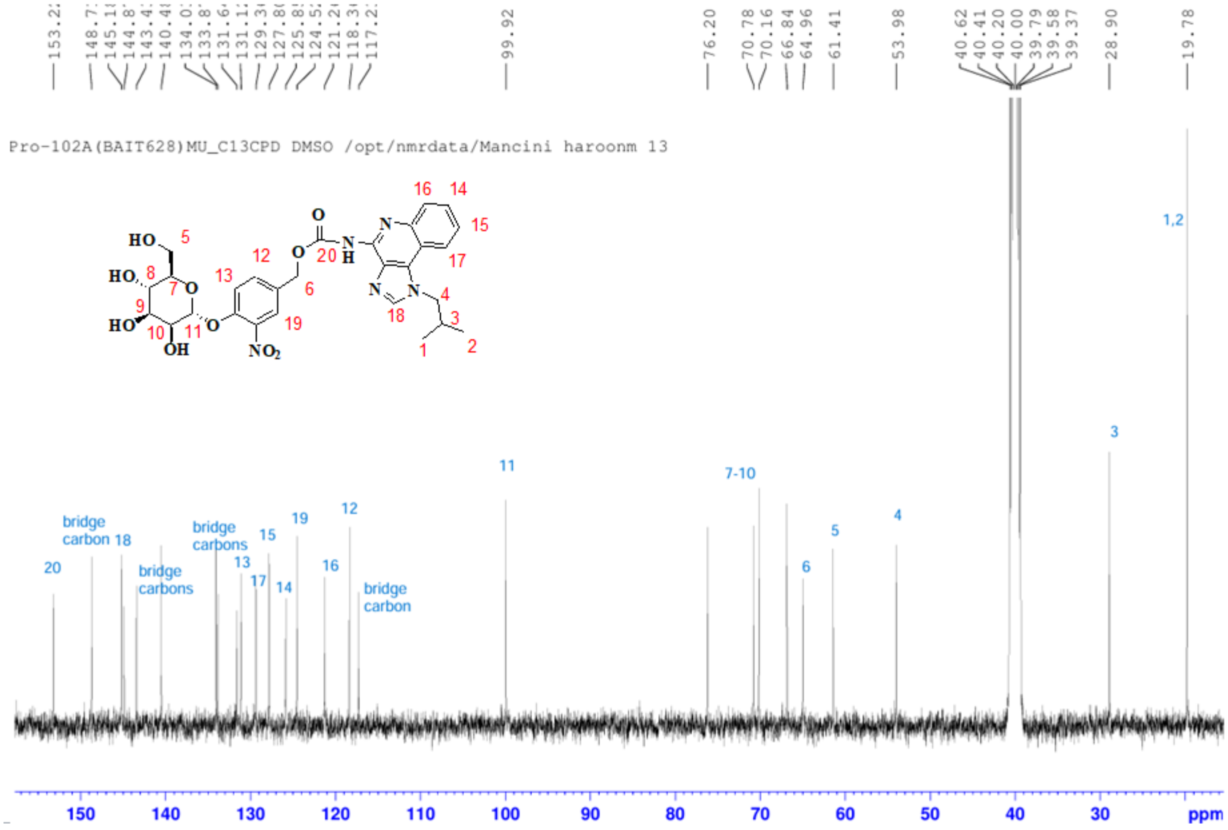

Figure S8: ^{13}C-NMR of BAIT 628

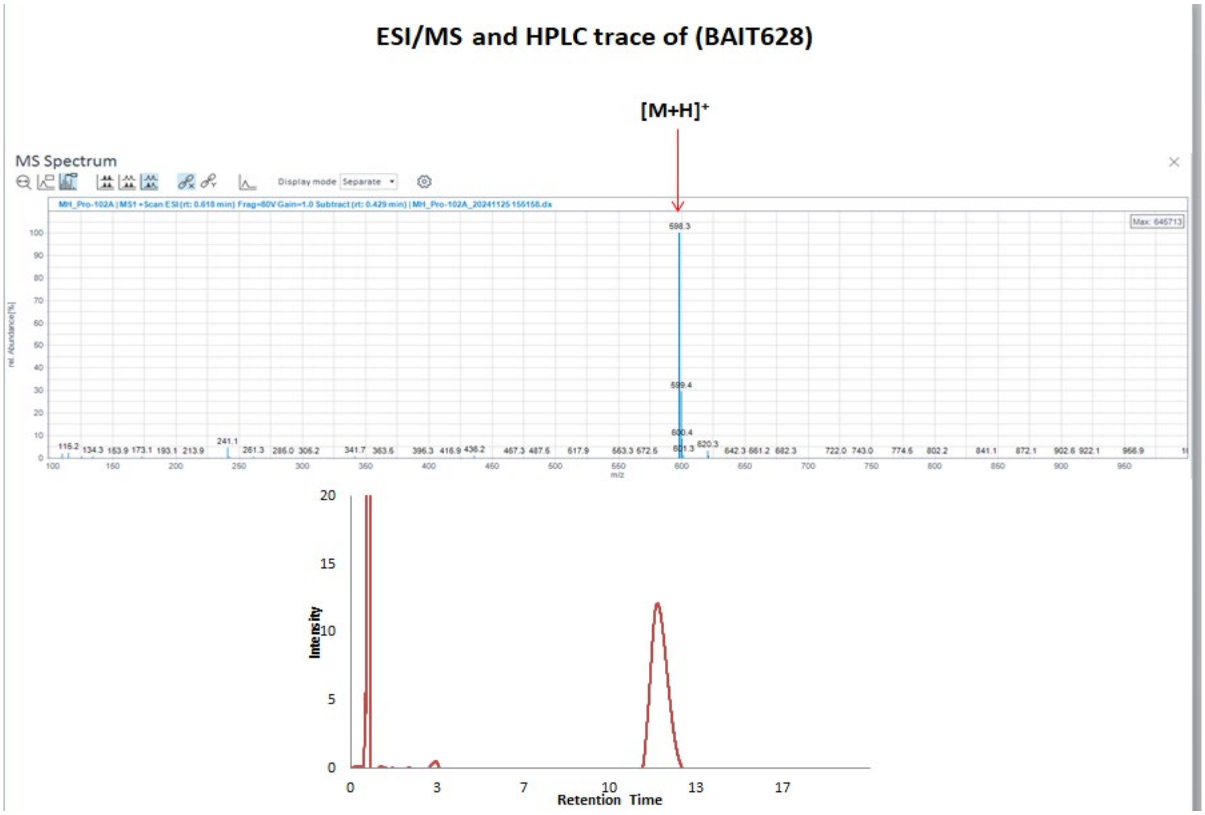

Figure S9: LCMS of BAIT628

References:

1. Ryan, A.T.; Pulukuri, A.J.; Davaritouchaee, M.; Abbasi, A.; Hendricksen, A.T.; Opp, L.K.; Burt, A.J.; Nielsen, A.E.; Mancini, R.J. Comparing the Immunogenicity of Glycosidase-Directed Resiquimod Prodrugs Mediated by Cancer Cell Metabolism. *Acta Pharmacol Sin* **2020**, *41*, 995–1004, doi:10.1038/s41401-020-0432-4.
